# Lesion Sterilization and Tissue Repair-Current Concepts and Practices

**DOI:** 10.5005/jp-journals-10005-1555

**Published:** 2018-10-01

**Authors:** Shaniya Sain, Reshmi J, Anandaraj S, Sageena George, Jyoti S Issac, Sheen A John

**Affiliations:** 1 Reader, Department of Pedodontics and Preventive Dentistry, PMS College of Dental Science and Research, Kerala, India; 2 Senior Lecturer, Department of Pedodontics and Preventive Dentistry, Golden Hills, Vattappara P.O, Kerala, India; 3 Professor, Department of Pedodontics, Golden Hills, Vattappara P.O, Kerala, India; 4 Professor, Department of Pedodontics and Preventive Dentistry, Golden Hills, Vattappara P.O, Kerala, India; 5 Reader, Department of Pedodontics and Preventive Dentistry, Golden Hills, Vattappara P.O, Kerala, India; 6 Reader, Department of Pedodontics and Preventive Dentistry, Golden Hills, Vattappara P.O, Kerala, India

**Keywords:** Lesion sterilization and tissue repair, Triple-antibiotic paste, 3-Mix paste

## Abstract

A deciduous tooth affected by dental caries involving the pulp tissue with subsequent periradicular pathosis at times makes the conventional endodontic procedures a dilemma for a variety of reasons. In this situation, lesion sterilization and tissue repair (LSTR) stands out as the only option by which dentists could increase the longevity of the deciduous teeth of a young child. This therapy aims to eliminate bacteria from the root canals by sterilizing the lesion and promoting tissue repair and regeneration by the host’s natural tissue responses. This article reviews the rationale, indications, contraindications and the procedure in detail.

**How to cite this article:** Sain S, Reshmi J, Anandaraj S, George S, Issac JS, John SA. Lesion Sterilization and Tissue Repair-Current Concepts and Practices, Int J Clin Pediatr Dent. 2018;11(5):446-450.

## INTRODUCTION

In routine dental practice, clinicians come across a number of cases where a deciduous tooth is affected by dental caries followed by periradicular change and are beyond conservation by traditional endodontic procedures.^1^ In such a situation, extraction becomes the only indicated treatment option. Pulp therapy at times become contra-indicated or compromised by factors like extensive root resorption, inadequate bone, and periodontal support, a child belonging to pre-cooperative age group etc. Then, pulp therapy becomes an enigma for the dentist.

But the premature loss of primary teeth can bring about disturbances like ectopic eruption, alteration in eruption sequence, space loss, development of deleterious habits, functional and speech impairment. Primary teeth play an integral role in the development of occlusion.^2,3^ Successfully disinfected and restored primary teeth can serve as the most promising space maintainer. The typical tooth morphology of primary teeth is that it has tortuous root canals, ramifications, the presence of multiple accessory canals, and ample medullary bone spaces that favor spreading of infection. To obtain a hermetic seal in the primary is complicated due to the lack of apical closure owing to physiologic root resorption of the primary tooth. The proximity of the developing permanent tooth germ to the roots of the primary teeth is another obstacle in endodontic therapy.^1,4,5^

In the modern era, a new perspective which is less invasive and less time-consuming procedure could be a ray of hope for the patient as well as the clinician. The LSTR-claims its significance in such clinical cases. The Cariology Research Unit of Niigata University School of Dentistry has developed the concept of LSTR therapy by Hoshino in 1990 and popularized by Takushige. The LSTR is an endodontic treatment procedure that involves non-instrumentation or minimal instrumentation followed by placement of antibiotic mixture in a propylene glycol vehicle to disinfect root canal systems, and peri-apical lesions. The basic concept of LSTR is that “do not remove or touch and leave it.” It medicates and treats caries, pulpitis and root canal infection. The principle behind LSTR is repair by natural defense mechanisms of host. Sterilizing the canals and pulp chamber by medicaments can decrease the bacterial load. Sterilization with medicaments will lead to 20 to 40% cleansing action and debridement. Most commonly a combination of three antibiotics along with solvent macrogol and propylene glycol are used, so it is also known as three mix MP pastes. If the procedure is successful tissue repair can be expected.^1^

Hoshino et al. in 1990 used combinations of antibiotics like metronidazole 500mg, ciprofloxacin 200 mg, minocycline 100 mg in 1:1:1 ratio.^6^ Takushige et al. in 1998 used the above antibiotics in the ratio 1:3:3.^1^ Met-ronidazole belonging to the nitroimidazole group, it binds to DNA and acts against gram positive and gram negative anaerobes. Ciprofloxacin is categorized under fluoroquinolone group acts by the inhibition of DNA Gyrase and facilitates the destruction of gram-negative organisms. Minocycline is a broad spectrum antibiotic that acts by inhibiting protein synthesis, collagenases, and matrix metalloproteinase. It destroys gram-positive as well as gram-negative microorganisms and also Spirochetes.^2,6-8^ Discolouration of teeth being a disadvantage of minocycline, antibiotics like amoxicillin, cefaclor, cefroxadine, fosfomycin or rokitamycin can be used as alternatives. Discoloration is due to the photo-induced reaction. Minocycline forms insoluble complexes by chelation reactions with calcium ions.^6-9^

Other combination pastes which were used are Grossman’s polyantibiotic paste (Penicillin, Bacitracin, and Streptomycin) and Ledermixpaste (Triamcinolone and Demeclocycline), Calcium hydroxide pastes, chlorhexi-dine paste, neomycin, polymyxin, and nystatin.^10^

Organic solvents like macrogol or propylene glycol are specifically used because of their increased penetration into the dentinal tubules.^11^

## PREPARATION OF TRIPLE ANTIBIOTIC PASTE

The most important step in LSTR is the preparation of triple antibiotic paste. The most common combination is the one proposed by Takushige et al. which includes metronidazole, ciprofloxacin, and minocycline. The commercially available antibiotics are taken in separate dappen dishes. The enteric coating of the tablet is removed by scraping the coating with a blade, and for the capsule the outer capsular material is removed. Then each of the components is powdered separately in clean mortar and pestle. Care should be taken to avoid wetting of powder. At this stage, if the powder has to be stored, it can be stored separately in tightly capped porcelain containers and stored in dark place or in the refrigerator to prevent exposure to light and moisture. After proper pulverization, each of the components is taken in a clean glass slab/mixing pad. Then a part of the solvent is dispensed. The triple antibiotic mixture has maximum effect when seven parts of powder are mixed with one part of solvent. So after dispensing, the powder is divided into seven parts and each part mixed separately with the solvent to ensure uniform consistency of the mix. The final preparation will be a soft ball-like structure of 1 mm diameter. If the mix is soft add more three mix powder to this. If the preparation becomes flaky, dry and too hard, then add more solvent. Resultant opaque paste has to be stored in airtight containers. If the mix turns translucent on storage, it has to be discarded.^10-12^

## PROCEDURE

After preparation of triple antibiotic paste, the next step is access cavity preparation. Local anesthesia is given, and rubber dam isolation is done. The access cavity is prepared using round bur, and the necrotic tissue is removed followed by irrigation with saline and sodium hypo-chlorite. The ethylenediaminetetraacetic acid (EDTA) is a better choice as it removes the smear layer leading to clean and patented dentinal tubules, which could allow deeper penetration of antibiotics into the dentinal tubules. If hemorrhage is present, it can be controlled by using sodium hypochlorite, an effective hemostatic agent. It does not interfere with pulpal healing and clots can be removed moreover it stops the pulpal hemorrhage that compromises pulpal healing and is nontoxic to pulpal tissue. Next step is the preparation of medication cavity using a round bur at the canal orifice which is 2 mm deep, and 1 mm wide, and is meant for retaining the triple antibiotic paste at the canal orifice. After proper drying, the paste is placed in the cavity, and permanent restoration is done using glass ionomer cement followed by stainless steel crown placement.^1^ In an *in vivo* study conducted by Prabhakar et al. on 60 infected primary molars, even though the teeth with root canals left unprepared also exhibited a clinical and radiographic success, the success rates were more for the biomechanically prepared (BMP) ones than the others.^12^

## FACTORS INFLUENCING THE ACTION OF TRIPLE ANTIBIOTIC PASTE

The quantity of drug should be sufficient enough to diffuse periapically from the canal and produce sterilization. The root canal infection has an array of aerobic and anaerobic flora, and this necessitates a combination of antibiotics rather than a single medicament to achieve complete sterilization of the canal. The use of drug combinations also prevents the development of resistant bacterial strains. When penicillin containing polyantibiotic paste was initially used it produced sensitization and superinfections. The medicaments used in root canals should cause the least damage to the host cells in spite of possessing antimicrobial properties, and should not cause any sensitivity/allergy to the patient. The medicaments used should be biocompatible.^10^

Smear layer acts as a barrier to the diffusion of triple antibiotic paste. Removal of the smear layer helps in proper absorption of medicaments, bringing about sterilization and disinfection. This can be achieved either by using EDTA or ultrasonic cleansing or both which can open up the dentinal tubules and increase penetration.^10^

Minocycline causes discoloration to the tooth. Sato et al. and Banchs and Trope suggested two methods to prevent this, but even these only reduce and not totally prevent discoloration. The first method is the application of bonding agent and curing it for 20 seconds and the second method is to use flowable composite and light cure it for 30 seconds.^8,13^

The LSTR helps to safeguard the deciduous tooth until its exfoliation, reducing the need for unnecessary extraction and placement of a space maintainer. The existence of accessory canals and the porosity and permeability of the pulpal floor region in deciduous teeth indicate a plausible association between pulpal and periodontal tissues. The triple antibiotic paste can be easily distributed through these areas and induce a sterile zone, which is expected to promote tissue repair.^14^

### Few Studies Regarding LSTR

Takushige et al. evaluated the effectiveness of 3-mix with MP paste and of a root canal sealer on the clinical outcome of LSTR in 56, 4 to 18-years-old patients. Out of 87 primary teeth, the clinical symptoms disappeared in 83 teeth, but in four cases it resolved only after retreat-ment using the same procedure. Draining sinuses and gingival abscesses disappeared after a few days, and the permanent successor erupted without any problems. The mean function time of the teeth was 680 days, except for one case with congenitally missing permanent teeth. All the cases were evaluated as successful.^1^

Prabhakar et al. studied the clinical and radiographic success of infected primary teeth using the triple antibiotic paste. They treated 60 teeth in two groups 1-year follow-up showed considerable clinical success in both groups, but a statistically significant difference was noted wherein the pulpectomy teeth had 83% success compared to 37% for pulpotomies.^12^

Jaya et al. evaluated and compared the clinical and radiographic effectiveness of ciprofloxacin, minocycline, metronidazole combination with ciprofloxacin, mino-cycline and tinidazole combination in 30 infected teeth and after 24 months follow up concluded that primary teeth with the peri-radicular lesions, can be conserved by using a combination of ciprofloxacin, minocycline and tinidazole antibacterial drugs.^15^

Burrus reported a case series of three cases using triple antibiotic paste, and he got positive results in all cases with the effective healing of periradicular tissue.^16^

Trairatvorakul evaluated the clinical and radiographic success rates of three mixed antibiotics in the non-instrumentation endodontic treatment of primary man-dibular molars at 24 to 27 months in 58 children. He conclude that non-instrumentation endodontic treatment using 3-mix-MP showed good clinical success but had a low success rate based on radiographic evaluation at 2-year follow-up. Hence, 3 Mix antibiotic treatment cannot replace a conventional root canal treatment agent as a long-term therapy.^17^

Nakornchai compared the clinical and radiographic success of 3Mix and Vitapex for root canal treatment of pulpally involved primary molars in 37 children he concluded that 3Mix can be used as a root canal treatment agent in pulpally involved primary teeth.^18^

Pinky et al. conducted a study on 4 to 10 year-olds with 40 infected primary teeth to evaluate the clinical and radio-graphic success of endodontic treatment using combinations of antibacterial drugs consisting of 3 mix (group A) and ciprofloxacin, ornidazole, and minocycline (group B). The result showed no statistically significant differences between the groups, suggesting 100% success with these combinations in treating necrosed primary teeth.^19^

Agarwal et al. assessed the clinical efficacy of pulp-otomy procedures using Pulpotec, LSTR using 3 mix, and zinc oxide eugenol (ZOE) pulpectomy. 60 pulpally involved teeth were selected and randomly divided into three groups. Clinical evaluation at 1 month showed only 70% success in the LSTR group (group 3) compared to 100% success in the other two groups. By the end of 12 months, the LSTR group displayed a poorer success rate compared to the ZOE pulpectomy group.^20^

*Enterococcus faecalis* the main endodontic pathogen in canals as well as peri-radicular tissues is highly virulent. Enterococcus groups were viable even after copious irrigation was performed. Hence; Alam et al. studied by in-vitro methods the susceptibility of *E. faecalis* to the triple antibiotic paste. He concluded that the 3-mix, at 100 ug/mL, is completely capable of inhibiting the growth of all strain when combinations was used.^21^

## INDICATIONS

There are an array of clinical conditions like nonvital teeth, advanced root resorption, strategically important teeth, severe bone loss and mobility, radiolucency in the furcal area, uncooperative patients, parents not willing for extraction and the like which can be considered to be treated with LSTR.^16,22^

## CONTRAINDICATIONS

The LSTR should not be a choice in children with known allergy to the agents used, radiographic evidence of excessive internal and external resorption, primary tooth nearing exfoliation and cases with perforated pulpal floor. LSTR is not recommended in children with infective endocarditis.^14^

## ADVANTAGES

The main advantage of LSTR is that it can be completed in one visit. Also, it is simple, painless, time-saving, and with less of a burden to patients both physically and psychologically. This is especially important when it comes to the management of pediatric patients. Bone regeneration occurs in cases were LSTR, was done.^17^

## DISADVANTAGES

The use of systemic antibiotics for local application raises concerns due to its adverse effects. The first and foremost disadvantage is that minocycline can cause tooth discoloration. As a remedy for the same, thibodeau and trope suggested cefaclor be used instead of minocycline.^13,23^ Also the radiolucent appearance of the triple antibiotic paste makes it difficult to assess the quality of the filling. To compensate for the same, iodoform could be added to impart radioopacity.^16^

These concerns of triple antibiotic paste include allergic reactions, the probability for the emergence of antibiotic-resistant bacterial strains, drug side effects, the risk of developmental anomalies in permanent teeth if used in primary teeth and cyst formation if the focus of chronic infection is left. The positive side to the above-said statement is the volume of these drugs used in LSTR is minimal, and there are no reported side effects.^14^

Concern regarding the hollow tube effect subsists in LSTR treated teeth because of the unfilled roots. The root canal that are not filled could be infused with tissue fluids; further, it gets stagnated and ultimately form a nidus for infection; whether this occurs in all cases or could be suppressed by host immunity is yet to be determined.^14^

## FUTURE PERSPECTIVE

The effects are ambiguous in relation to the damage the procedure may cause to the underlying tooth bud, its effect on the growth and development in children, the necessity for the concurrent usage of systemic antibiotics and so on. Thus for prudent case selection and more effective management, long-term studies are recommended in future.

## CONCLUSION

Root canal complexity of the primary teeth is well known. Compounding to this, are the presence of thin dentinal walls, flaring deciduous roots, the presence of resorbing apices that may cause an undue effect of the irrigant used, non-negotiable tortuous canals, and uncooperative children and these may prompt clinicians for an option other than untimely extraction. In this regard, LSTR could be considered as an alternative that eliminates the causative microorganisms from the lesion by sterilizing and helping repair with the aid of individual’s own natural immune system.
